# Possible increased risks of cholecystitis in patients with idiopathic pulmonary fibrosis treated with nintedanib

**DOI:** 10.1007/s00228-025-03867-x

**Published:** 2025-07-23

**Authors:** Catherine Kiani, Alexandre Bleinc, Theodora Bejan-Angoulvant, Marion Teulier, Sylvain Marchand-Adam

**Affiliations:** 1 Service of Pneumology and Functional Respiratory Explorations, Hôpital Bretonneau, CHRU de Tours, 10 Bd Tonnellé, Cedex 1, 37032 Tours, France; 2 Centre for the Study of Respiratory Pathology (CEPR) INSERM U1100, Faculty of Medicine, University of Tours, Tours, France; 3 Respiratory Service, CH Blois, Blois, France; 4 University of Tours, EA 4245 Tours, France; 5 Clinical Pharmacology Department, CHRU de Tours, Tours, France

Nintedanib, a tyrosine kinase inhibitor (TKI), is an antifibrotic agent currently recommended for patients with mild to moderate idiopathic pulmonary fibrosis(IPF). It inhibits key growth factors implicated in the pathogenesis of IPF such as vascular endothelial growth factor receptor (VEGF-R) [1]. The summary of product characteristics for nintedanib does not describe the existence of cholecystitis as a documented side effect [2]. We conducted a retrospective analysis of cholecystitis cases in 176 patients receiving care for IPF at the University Hospital of Tours from January 2007 to February 2021. The study was approved on November 2, 2023, by the Institutional Review Board of the Société de Pneumologie de Langue Française (CEPRO 2023–034). We excluded 12 patients that had undergone cholecystectomy prior to IPF management. Among the remaining 164 patients, 69 patients never received antifibrotic agents, 35 patients received only nintedanib, 20 received only pirfenidone, and 40 received both antifibrotic agents sequentially. During the 7081 months of follow-up for the 164 patients with IPF, 5 cases of cholecystitis occurred during the 1470 months of treatment with nintedanib, 0 cases during the 1144 months of treatment with pirfenidone, and only 1 case during the 4467 months of follow-up without antifibrotic treatment. All 5 patients who developed cholecystitis while taking nintedanib (Table 1) were male, with a median age of 78 years [63; 86], a median forced vital capacity (FVC) of 69% [44; 77], and a median diffusion capacity of carbon monoxide (DLCO) of 28% [5; 48]. Prior to the episode of cholecystitis, all five patients had undergone regular liver function tests (every 3 months), all of which were normal. Among the usual risk factors for cholecystitis, only two patients were overweight, and one other had recently experienced significant weight loss (> 5 kg in 4 months). Two patients underwent initial cholecystectomy. One had a favorable outcome with a delayed scar healing. The other experienced severe infectious complications (hepatic abscess, acute pancreatitis, *Enterococcus faecium* bacteremia) followed by an IPF exacerbation. The remaining patients received initial medical therapy. One had a favorable outcome, one developed acute pancreatitis and underwent secondary cholecystectomy. The third patient developed severe bacteremia. Due to the respiratory condition’s severity, surgery was not possible, and the patient died.

**Table 1:** The 5 cases of cholecystitis in IPF patients treated with nintedanib followed at the UHT

	Sex^a^	Age at cholecystitis diagnosis^b^	Therapy duration with nintedanib^c^ (dose^d^)	FVC^e^	DLCO^f^	First line cholecystitis treatment^g^	Lithiasis^h^	Outcome^i^
Patient 1	M	72	40 (300)	44%	5%	Medical	No	*K. oxytoca* and *C. perfringens* bacteremia, percutaneous drainage, respiratory deterioration, death
Patient 2	M	83	31 (300)	69%	25%	Medical	Yes	Acute pancreatitis, subsequent cholecystectomy
Patient 3	M	63	7 (300)	77%	48%	Laparoscopic cholecystectomy	Yes	Delayed wound healing
Patient 4	M	86	32 (200)	69%	28%	Laparotomy cholecystectomy	Yes	Hepatic abscess, acute pancreatitis, *E. facium* bacteremia, respiratory deterioration
Patient 5	M	78	49 (300)	77%	31%	Medical	Yes	Favorable outcome

^a^M = male, F = female

^b^Age in years at the time of cholecystitis diagnosis

^c^Cumulative duration of nintedanib treatment at the time of cholecystitis diagnosis, expressed in months

^d^Dose of nintedanib at the time of cholecystitis in mg/day

^e^Last available FVC on the patient’s pulmonary function tests (PFTs) prior to the cholecystitis episode, expressed as a percentage of predicted value

^f^Last available DLCO on the patient’s PFTs prior to the cholecystitis episode, expressed as a percentage of predicted value

^g^First-line treatment of cholecystitis; if the treatment was medical, it included at least one antibiotic targeting the digestive germs

^h^The presence or absence of lithiasis was proved or disproved by CT scan

^i^Patient’s outcome after first-line treatment

Additionally, we examined the Food and Drug Administration Adverse Event Reporting System (FEARS). We extracted reports of biliary pathologies occurring in IPF patients treated with nintedanib or pirfenidone during the period between their market availability in the USA and March 1, 2020. The keywords employed for extraction included “Gallbladder,” “Cholecystitis,” “Cholelithiasis,” “Cholangitis,” “Cholestasis,” “Cholelithotomy,” and “Cholecystectomy.” We extracted 104 biliary pathology reports out of 9713 adverse events reported with nintedanib (1.07%), in contrast to 64 reports out of 18,729 adverse events reported with pirfenidone (0.34%), ROR = 3.16, 95% CI (2.31–4.31). Instances of death attributable to biliary disease were observed in 15 patients treated with nintedanib compared to 4 patients treated with pirfenidone, ROR = 7.24, 95% CI (2.40–21.82).

In vivo toxicity studies, the bile duct has been identified as one of the target organs affected by nintedanib toxicity [3]. Monkeys exposed to high doses of nintedanib exhibited the presence of inflammatory cells in the gallbladder upon microscopic examination. In a mice exposure study, treatment-related fibrosis and ulceration were observed in the gallbladder. Rat studies demonstrated inflammation and hyperplasia of the ductal epithelium in the main bile duct. The INMARK study reported a greater increase in bilirubin levels and notably one case of cholecystitis in patients with nintedanib treatment compared to the placebo group [4].

Cholecystitis is listed as an adverse event in the summaries of product characteristics for other TKIs, including sunitinib, lenvatinib, sorafenib, and axitinib. Numerous cases of alithiasis attributed to these compounds have been described [5, 6]. While all these inhibitors, including nintedanib, are multikinase inhibitors, they share the VEGF-R as a common target. There is speculation that acute cholecystitis might be instigated by an antivascular effect [7]. The inhibition of the VEGF receptor could disturb the homeostasis between the endothelium and platelets due to TKI-induced endothelial cell damages, potentially leading to microthrombi formations and ischemia [8]. These events might be exacerbated by nintedanib accumulation in the gallbladder lumen [2].

Our data therefore suggest a possible increased risk of cholecystitis in patients treated with nintedanib. These cholecystitites could be accompanied by an increased risk of morbidity and mortality given the patient profile for this treatment. A study of larger cohorts of patients treated with antifibrotic agents is needed to confirm our results. This adverse effect should be included in the product characteristics for nintedanib.

## Data Availability

No datasets were generated or analysed during the current study.

## Abbreviations

*DLCO*: Diffusion capacity of carbon monoxide
*FEARS*: Food and drug administration adverse event reporting system
*FVC*: Forced vital capacity
*IPF*: Idiopathic pulmonary fibrosis
*TKI*: Tyrosine kinase inhibitor
*UHT*: University hospital of tours
*VEGF-R*: Vascular endothelial growth factor receptor
